# Efficient Delivery of DNA Using Lipid Nanoparticles

**DOI:** 10.3390/pharmaceutics14081698

**Published:** 2022-08-15

**Authors:** Lishan Cui, Serena Renzi, Erica Quagliarini, Luca Digiacomo, Heinz Amenitsch, Laura Masuelli, Roberto Bei, Gianmarco Ferri, Francesco Cardarelli, Junbiao Wang, Augusto Amici, Daniela Pozzi, Cristina Marchini, Giulio Caracciolo

**Affiliations:** 1 School of Biosciences and Veterinary Medicine, University of Camerino, 62032 Camerino, Italy; 2 NanoDelivery Lab, Department of Molecular Medicine, Sapienza University of Rome, 00161 Rome, Italy; 3 Institute of Inorganic Chemistry, Graz University of Technology, 8010 Graz, Austria; 4 Department of Experimental Medicine, “Sapienza” University of Rome, 00161 Rome, Italy; 5 Department of Clinical Sciences and Translational Medicine, University of Rome “Tor Vergata”, 00133 Rome, Italy; 6 National Enterprise for NanoScience and NanoTechnology (NEST), Scuola Normale Superiore, 56127 Pisa, Italy

**Keywords:** DNA vaccines, lipid nanoparticles, HER2

## Abstract

DNA vaccination has been extensively studied as a promising strategy for tumor treatment. Despite the efforts, the therapeutic efficacy of DNA vaccines has been limited by their intrinsic poor cellular internalization. Electroporation, which is based on the application of a controlled electric field to enhance DNA penetration into cells, has been the method of choice to produce acceptable levels of gene transfer in vivo. However, this method may cause cell damage or rupture, non-specific targeting, and even degradation of pDNA. Skin irritation, muscle contractions, pain, alterations in skin structure, and irreversible cell damage have been frequently reported. To overcome these limitations, in this work, we use a microfluidic platform to generate DNA-loaded lipid nanoparticles (LNPs) which are then characterized by a combination of dynamic light scattering (DLS), synchrotron small-angle X-ray scattering (SAXS), and transmission electron microscopy (TEM). Despite the clinical successes obtained by LNPs for mRNA and siRNA delivery, little is known about LNPs encapsulating bulkier DNA molecules, the clinical application of which remains challenging. For in vitro screening, LNPs were administered to human embryonic kidney 293 (HEK-293) and Chinese hamster ovary (CHO) cell lines and ranked for their transfection efficiency (TE) and cytotoxicity. The LNP formulation exhibiting the highest TE and the lowest cytotoxicity was then tested for the delivery of the DNA vaccine pVAX-hECTM targeting the human neoantigen HER2, an oncoprotein overexpressed in several cancer types. Using fluorescence-activated cell sorting (FACS), immunofluorescence assays and fluorescence confocal microscopy (FCS), we proved that pVAX-hECTM-loaded LNPs produce massive expression of the HER2 antigen on the cell membrane of HEK-293 cells. Our results provide new insights into the structure–activity relationship of DNA-loaded LNPs and pave the way for the access of this gene delivery technology to preclinical studies.

## 1. Introduction

**Figure 1:**
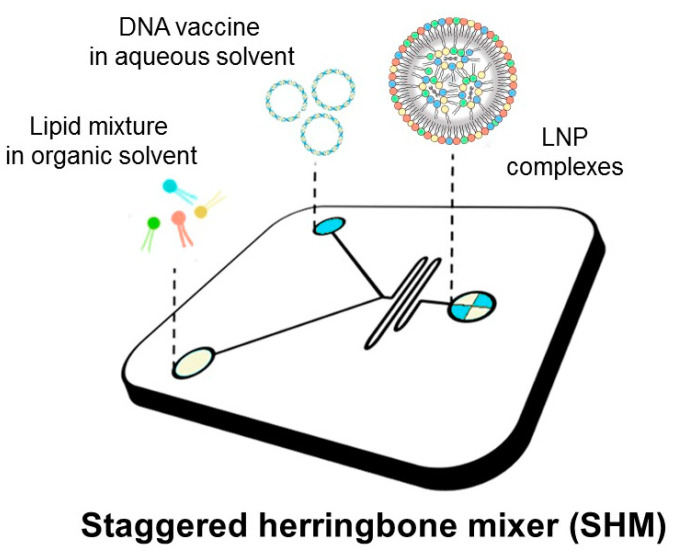
Schematic representation of the synthesis procedure of LNP DNA vaccines.

Despite the massive involvement of resources in cancer studies and in the development of potential therapeutic strategies, cancer is still one of the main causes of death worldwide [1]. The conventional approaches in cancer therapy, such as chemotherapy, radiotherapy, and surgery, are often effective in early-stage tumors but not in those diagnosed in advanced stages. The temporary suspension of screening programs caused by the COVID-19 pandemic exacerbated this trend [2]. The non-specific targeting of both tumoral and healthy cells observed in traditional chemotherapies causes side effects that have significantly evolved over the last decades [3]. Recently, immunotherapy has been investigated as an alternative strategy to most traditional therapies. It aims at boosting patient immunity or promote tumor-cell killing by targeting specifically immunomodulator pathways [4]. Vaccination, one of the most successful strategies for the prevention of infectious diseases, emerged as an attractive immunotherapeutic approach against cancer [5]. DNA vaccines consist of plasmid DNA (pDNA) of bacterial origin which encodes for the target antigen that can elicit both humoral and cell-mediated immunity. At the formulation level, DNA vaccines hold several benefits over whole pathogen (i.e., attenuated and inactivated viral vaccines) or protein-based vaccines, such as simple and rapid manufacturing processes or the easy manipulation of the encoded antigen by DNA engineering [6]. Additionally, the thermostability of DNA vaccines tackles the complications linked to cold chain [7] maintenance, which instead is essential for less stable vaccines (e.g., mRNA-based vaccines) to avoid the inactivation during supply. Despite many clinical trials of DNA vaccines for cancers now being underway, none have been licensed for human use thus far. The biggest hurdle for their clinical translation is the difficulty in efficient delivery to the cell machinery. Viral vectors are associated with many risks such as genome integration and possible host rejection. Nonviral DNA delivery systems employ either chemical or physical approaches. The most investigated physical method is electroporation, which is based on the application of a controlled electric field to enhance DNA penetration into cells and generates high levels of gene transfer in vivo [8]. However, despite its versatility and lower amount of required DNA, this method may cause cell damage or rupture, non-specific targeting, and even degradation of pDNA. Conversely, nanoparticles (NPs) hold great promise for efficient DNA delivery. Such nanodelivery systems protect the nucleic acid payload from degradation, favor versatile formulation strategies to cross the barriers of cell internalization, enhance specific immune cell targeting by surface modifications, and exploit pH-sensitive shells to improve endosomal escape. Cationic lipid–DNA complexes (lipoplexes) have been largely employed as gene delivery systems since cationic lipids allow an easy complexation with negatively charged pDNA. The conventional method for manufacturing lipoplexes is the bulk mixing process that leads to heterogeneous large-size vesicles and requires an additional down-sizing technique (i.e., sonication or extrusion) to achieve the desired particle size [9]. To overcome this limitation, microfluidic mixing has emerged as a robust, scalable, and highly reproducible technique to encapsulate gene therapeutics. The result of the mixing process is a lipid NP (LNP) with the genetic material encapsulated in the lipid core [9]. LNPs with different physicochemical characteristics can be achieved by adjusting several factors, such as (i) lipid composition, (ii) total lipid/DNA weight ratio, (iii) cationic lipid/DNA weight ratio, (iv) solution concentration, and (v) microfluidic operating parameters, such as the flow rate ratio (FRR) or the total flow rate (TFR) [10]. In this work, we used a microfluidic platform to generate DNA-loaded LNPs (as depicted in Figure 1) with distinct physical–chemical properties as determined by dynamic light scattering (DLS), synchrotron small-angle X-ray scattering (SAXS), and transmission electron microscopy (TEM). Previous investigations have demonstrated that multicomponent lipoplexes overcome binary lipoplexes in transfection efficiency (TE) due to a peculiar endosomal escape ability [11,12,13,14]. According to these findings, here we prepared multicomponent LNPs made of two cationic and two neutrally charged lipids.

For in vitro screening, LNPs were administered to human embryonic kidney 293 (HEK-293) and Chinese hamster ovary (CHO) cell lines and ranked for their TE and cytotoxicity. The most promising LNP formulation was loaded with the DNA vaccine pVAX-hECTM targeting the human neoantigen HER2, an oncoprotein that is overexpressed in several cancers and that represents an ideal immunogenic target for tumor vaccines [15]. Combing results from fluorescence-activated cell sorting (FACS), immunofluorescence assays, and fluorescence confocal microscopy (FCS) we demonstrated that optimized LNPs efficiently deliver pVAX-hECTM into HEK-293 cells, leading to massive expression of the HER2 antigen on the cell membrane. Our results pave the way for the development of LNP DNA vaccines and immunotherapies against cancer and other diseases.

## 2. Materials and Methods

### 2.1. Microfluidic Manufacturing of LNPs

The cationic lipids 1,2-Dioleoyl-3-trimethyl-ammonium-propane (DOTAP) and (3β-[N-(N’,N’-dimethyl-aminoethane)-carbamoyl])-cholesterol (DC-Chol), along with the two zwitterionic lipids dioleoyl phosphatidylethanolamine (DOPE) and 1,2-dioleoyl-sn-glycero-3-phosphocholine (DOPC) were obtained from Avanti Polar Lipids (Alabaster, AL, USA). The pDNA coding for firefly luciferase reporter gene (pmirGLO) was bought from (Promega, Madison, WI, USA). NanoAssemblr^®^ Benchtop from Precision NanoSystems Inc. (Vancouver, BC, Canada) equipped with a Y-shape staggered herringbone micromixer was used for lipid nanoparticle (LNPs) development. Lipids were individually dissolved in ethanol (100%) at a molar ratio of 25% mol for each lipid (Table 1) and a total concentration of 12.5 mM. The pDNA expressing luciferase was dissolved in sodium acetate 25 mM, pH = 4, to obtain a final concentration of 0.2 mg/mL and 0.1 mg/mL. The two solutions were mixed in the microfluidic chip at a fixed total flow rate (TFR; 2 mL/min) and a flow rate ratio (FRR) DNA/lipid of 3:1, reducing the ethanol concentration to 25% after the micromixing. For each DNA condition (0.2 mg/mL and 0.1 mg/mL), all formulations were produced at three different lipid/DNA weight ratios (Rw = 5, 10, and 20) hereafter referred to as LNP_5_, LNP_10_, and LNP_20_. The LNPs were subsequently purified by dialyzing against 500 mL of phosphate-buffered saline (PBS) at pH 7.4 with Slide-A-Lazer. Dialysis cassettes (0.5–3 mL, MWCO 3.5 kDa, Thermo Scientific, Rockford, AZ, USA) for 19 h. To evaluate particle stability, LNPs were stored at 4 °C for 15 days.

### 2.2. Plasmid Preparation

PmirGlo and the DNA vaccine pVAX-hECTM (encoding the human extracellular and transmembrane domains of the human HER2 receptor) were transformed into *E. coli* strain DH5alpha and grown in Luria–Bertani medium supplemented with kanamycin. DNA plasmids were purified using a Maxiprep kit (Qiagen, Hilden, Germany) and their concentration was determined spectrophotometrically at 260 nm.

### 2.3. Encapsulation Efficiency Measurements

pDNA encapsulation efficiency of LNP_10_ and LNP_20_ was measured by using a Quant-iT Pico-Green dsDNA assay kit (ThermoFisher Scientific, Waltham, MA, USA). All LNP samples were diluted 300-fold in TE buffer and placed on a Corning^®^ 96 Well Solid Polystyrene Microplate (Sigma-Aldrich, St. Louis, MI, USA). LNPs were lysed to release the encapsulated DNA through the addition of Triton X-100 (1% volume) to each well. Control samples were not lysed and consisted of free pDNA. The reagent Quant-iT PicoGreen was added to all wells and the samples were incubated for 5 min at room temperature. Fluorescence (excitation wavelength = 475 nm, emission wavelength = 500–550 nm) was measured by using a Glomax Discover System (Promega, Madison, WI, USA). Encapsulation efficiency (EE%) was determined by measuring the fluorescence pre-lysis and post-lysis of LNPs and calculated following Equation (1):

(1) $$\%\;\mathrm{EE}=\frac{\left(\mathrm{Lysed}\;\mathrm{LNP}-\;\mathrm{not}\;\mathrm{lysed}\;\mathrm{LNP}\right)}{\mathrm{Lysed}\;\mathrm{LNP}}\times 100$$

The DNA concentration in μg/mL of LNPs was estimated using a calibration curve obtained from measurements of pDNA at different known concentrations (Figure S1 in the Supplementary Materials).

### 2.4. Size and Zeta-Potential Experiments

Size and zeta-potential measures were made at 25 °C by using Zetasizer Nano ZS90 (Malvern, UK). LNPs were diluted with distilled water 1:100 before the measurement. Results for size and zeta-potential of three replicates are reported as mean ± standard deviation. For particle size, we report the Z-average that is the intensity weighted mean hydrodynamic size of the particles measured by dynamic light scattering (DLS).

### 2.5. Synchrotron Small-Angle X-ray Scattering

Synchrotron small-angle X-ray scattering (SAXS) investigations were performed at the Austrian SAXS beamline at ELETTRA (Trieste, Italy). The calibration of the detector (Pilatus3 1 M, Dectris, Baden, Switzerland) was made by using silver behenate powder (d-spacing = 58.376 Å), q-range settled within 0.05 and 1.5 nm^−1^, through 10 s X-ray exposure (no radiation damage was detected). The temperature was monitored in the vicinity of the capillary. The corrections for background, primary beam intensity, and detector efficiency were all included in the analysis of SAXS patterns.

### 2.6. Transmission Electron Microscopy

LNPs samples (8 μL) were placed on formvar–carbon-coated copper grids (EMS, Orefield, PA, USA) and let adsorb for 5 min. The resultant film was stained with a solution of 2% uranyl acetate at room temperature for 1 min. Staining solution excess was absorbed with the filter paper. Grids were air-dried for 1 h before the observation with TEM Morgagni 268D (Philips, Amsterdam, The Netherlands).

### 2.7. Cell Culture

The two cell lines, human embryonic kidney-293 (HEK-293) and Chinese hamster ovary (CHO), were purchased from American Type Culture Collection (ATCC, Rockville, MD, USA) and maintained in culture in Dulbecco’s Modified Essential Medium (DMEM, Gibco, Life Technologies, Carlsbad, CA, USA) enriched with 10% fetal calf serum (FCS, Gibco, Life Technologies) and 1% penicillin–streptomycin (Gibco, Life Technologies). Cells were maintained at 37 °C with 5% CO_2_ under a humidified atmosphere.

### 2.8. Transfection Efficiency Experiments

Cells (KEK-293 or CHO) were seeded on a 96-well plate (10,000 cells/well). After 24 h cells were treated with LNPs and lipofectamine^TM^ 3000 (Life Technologies, Carlsbad, CA, USA) with 0.33 μg pDNA/well in Optimem medium (Life Technologies, Carlsbad, CA, USA). This dose corresponds to a typical threshold for lipoplexes leading to high TE with a minor effect on cell viability [16]. After 3 h of incubation at 37 °C and 5% CO_2_, DMEM 20%FBS was added to each well. After 48 h, cells were washed in PBS 1× (Phosphate-buffer saline) and lysed using lysis buffer 1× 20 μL/well. Luciferase expression was analyzed by Luciferase Assay System (Promega, Madison, WI, USA) on half of the sample (10 μL), while half (10 μL) was used to evaluate the sample protein content through Pierce BCA Assay Protein Kit (Thermo Fisher Scientific, Waltham, MA, USA) the transfection efficiency (TE) was expressed as relative light units (RLU) per mg proteins.

### 2.9. Cell Viability Assay

The effects of LNP with respect to Lipofectamine^TM^ 3000 (Invitrogen, Waltham, MA, USA) on cell viability were evaluated by seeding 8000 HEK-293 and CHO cells/well in 96-well plates in a complete medium. The day after, appropriate concentrations of plasmid DNA (pmirGLO) encapsulated in LNP or complexed with lipofectamine were added. After 48 h, cell viability was determined using the MTT assay (Sigma Aldrich, St. Louis, MO, USA), which is based on the conversion of tetrazolium salt [3-(4,5-dimethylthiazol-2-yl)-2,5-diphenyl-2H-tetrazolium bromide] into formazan by means of mitochondrial enzymes. The formazan crystals were dissolved using dimethyl sulfoxide (DMSO) and the well absorbance was measured at 540 nm using Multiskan Ascent 96/384 Plate Reader. Each sample was tested with eight replicates, and the experiments were performed in triplicate. The cell viability was reported as percentage of viable cells in respect to control cells. Statistical analysis was performed using a one-way ANOVA analysis of variance and Bonferroni’s multiple comparison test. The data are represented as means ± SEM. The 95% confidence interval was used as the critical level for significance.

### 2.10. Confocal Microscope Experiments

Live-cell fluorescence imaging was performed with a Zeiss LSM 800 confocal microscope equipped with a 63X, 1.4 N.A. oil immersion objective and GaAsP detectors. Experiments were carried out at 37 °C and 5% CO_2_ using an incubation chamber. Approximately 10^5^ HEK or CHO cells were seeded in a cover glass multi-well (8 wells, Sarstedt) 24 h before the experiment. Cells were incubated with LNP–Texas Red 1X for 3 h then washed 2 times with PBS and labeled with 0.1 µL of 10 mg/mL Hoechst 33,342 (ThermoFisher). Hoechst was excited with 405 nm diode laser; Texas Red was excited with 561 nm HeNe laser and its emission collected in the 570–630 nm range; 1024 × 1024 pixel images were collected. For iMSD analysis on LNP-TexasRed 1X, time-lapse series of 500 frames (256 × 256 pixels, 50 nm/pixel) with a temporal resolution of 200 ms/frame were collected using the same excitation/emission parameters described above.

### 2.11. iMSD Analysis

iMSD analysis of the time-lapse movies was carried out with custom scripts working on MATLAB (MathWorks Inc., Natick, MA, USA), as described in detail in Refs [17,18]. Briefly, the spatiotemporal correlation function (STCF) was calculated for each time-lapse series; 2D-Gaussian fitting of STCF provides the iMSD curve, describing the ensemble diffusion law of imaged objects. Each iMSD curve was then fitted to extract the structural-dynamic parameters: diffusion coefficient and *y*-axis intercept value, with the latter related to average particle size.

### 2.12. Immunofluorescence Analysis

HEK-293 cells were plated in a 24-well plate (1 × 10^5^ cells/well). One day after plating, 70–90% of confluent cells were transiently transfected with 0.5 mg or 1 mg pVAX-hECTM encapsulated in LNP or complexed with Lipofectamine 3000, according to the manufacturer’s instructions. Forty-eight hours after transfection, cells were fixed for 5 min with phosphate-buffered saline (PBS)–4% paraformaldehyde (Sigma, St. Louis, MO, USA). After incubation in blocking buffer (PBS–10% bovine serum albumin (BSA; Sigma, Milan, Italy) for 20 min, cells were incubated for 1 h at 37 °C with the primary antibody trastuzumab (anti-human HER2 antibody, 1:50). After washing, cells were incubated with Alexa Fluor 488-conjugated anti-human IgG secondary antibody (Invitrogen Molecular Probes, Eugene, OR, USA) at a dilution of 1:200 for 1 h at 37 °C. Finally, cells were examined under Fluorescence Microscope (Carl Zeiss GmbH, Munich, Germany) to assess membrane expression of the oncoantigen HER2.

### 2.13. Flow Cytometry

HEK-293 cells were plated in a 6-well plate (1 × 10^6^ cells/well). One day after plating, 70–90% of confluent cells were transiently transfected with pVAX-hECTM encapsulated in LNPs. Forty-eight hours after transfection cells were separated into single-cell suspension and 1 × 10^6^ cells for each experimental condition were washed in staining buffer (0.1% NaN_3_, 2% FBS in PBS) and incubated with the primary antibody anti-HER2 (trastuzumab) at 4 °C for 1 h. After three washes, Alexa Fluor 488-conjugated anti-human IgG secondary antibody was added and cells were incubated for 1 h at 4 °C. Cells were washed and resuspended in PBS before the analysis performed using FACS equipped with Cell Quest software (BD Pharmingen, BD Life Sciences, San Jose, CA, USA). FlowJo software (BD Life Sciences, San Jose, CA, USA) was employed for data analysis.

## 3. Results and Discussion

**Figure 2:**
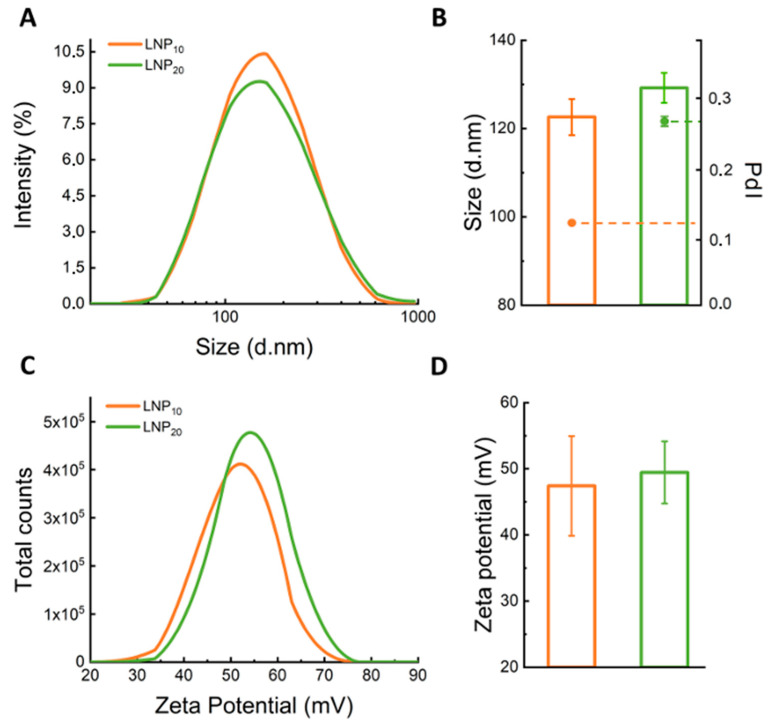
**Size and zeta-potential of LNPs**. (**A**) Size distributions, (**B**) mean size (reported as z-average) and polydispersity index (PdI) values, (**C**) zeta-potential distributions, and (**D**) average zeta-potential of LNP_10_ (orange) and LNP_20_ (green).

LNPs were produced at three different lipid/DNA weight ratios (i.e., Rw = 20, 10, and 5) and are hereafter referred to as LNP_5_, LNP_10_, and LNP_20_. The choice of preparing LNPs at different Rw values is because this is an influential parameter for the physical–chemical properties of lipid-based gene delivery systems [19]. Among them, lipoplexes are the gold standard in lipid-mediated gene transfection (lipofection) [20] and were used as a reference in the following. When lipoplexes are prepared at large lipid/DNA weight ratios (that typically occur for Rw > 10 [21]), the moles of cationic lipids exceed those of anionic DNA. Hence, lipoplexes are positively charged, and the net cationic charge makes them efficiently interact with the negatively charged plasma membrane [22]. On the other hand, at low lipid/DNA weight ratios (Rw < 5) the balance between positive and negative charges results in the formation of neutrally charged complexes of large sizes that are not compatible with gene delivery purposes [23]. However, the effect of Rw on the synthetic identity of LNPs has been poorly evaluated so far. Therefore, the first step of the present investigation was a thorough characterization of LNPs in terms of size, zeta-potential, and polydispersity index (PdI). As Table S1 shows, the large size (Z-average > 400 nm) and polydispersity (PdI > 0.9) of LNP_5_ made it unsuitable for gene delivery, and this formulation was therefore excluded from the following experiments. On the other side, as shown in the results reported in Figure 2, LNP_10_ and LNP_20_ exhibited positive zeta-potential, small sizes ranging from 120 to 130 nm, and adequate polydispersity index (PdI = 0.12 and 0.27, respectively). The DNA encapsulation efficiency for LNP formulations was larger than 60% (details are reported in Figure S1 in the Supplementary Materials).

The previous literature has recognized that the nanoscale structure of lipoplexes is critical for the mechanism by which they promote intracellular delivery of DNA [11], and the investigation of their structure–activity relationship has been the subject of many theoretical and experimental studies [12,24,25,26]. Mechanistic understanding of lipoplex–cell interactions led to the development of optimized formulations with superior TE even in hard-to-transfect cells [16] where the Lipofectamine^TM^ transfection gold standard failed. On the other side, this basic knowledge has not been developed for LNPs yet, but it is mandatory for the development of optimized formulations [27]. Most studies have explored the nanostructure of siRNA- and mRNA-LNPs by SAXS. While there is consensus that RNA is positioned within the particle interior, understanding of the nanoscale organization of lipids and RNA by SAXS remains elusive. Depending on compositional and operating factors, different arrangements have been reported, such as multilamellar structures [28], Ia3d and Pm3n cubic phases [29], and other non-lamellar phases where RNA molecules are confined within aqueous cylinders [30].

**Figure 3:**
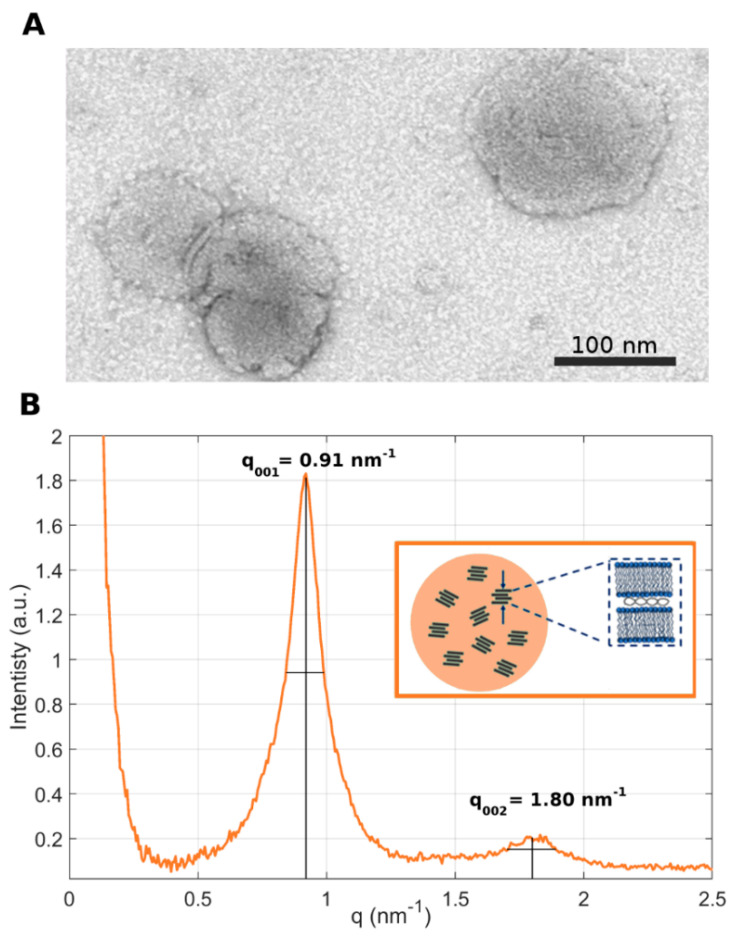
**Nanostructure of LNPs**. (**A**) Representative TEM image of LNP_10_ (panel A) (scale bar = 100 nm). (**B**) Synchrotron SAXS pattern of LNP_10_. The peaks arose from the lamellar periodicity of the system along the normal direction to the lipid bilayer. The small persistence length suggests that LNPs are made of randomly oriented lamellar domains, as schematically depicted in the inset. An average domain size made of 6 repeated units was estimated by relating the location of the first-ordered Bragg peak with its full width at half maximum (FWHM).

In the present investigation, the nanostructures of LNP_10_ and LNP_20_ were characterized by TEM and synchrotron SAXS. The representative TEM image reported in Figure 3A shows that LNPs are spherically shaped with a size around 100 nm, in agreement with the DLS results reported in Figure 2. Synchrotron SAXS curves exhibited two broad Bragg peaks, located at q_001_ = 0.91 nm^−1^ and q_002_ = 1.80 nm^−1^ ≈ 2 q_001_ (Figure 3B).

**Figure 4:**
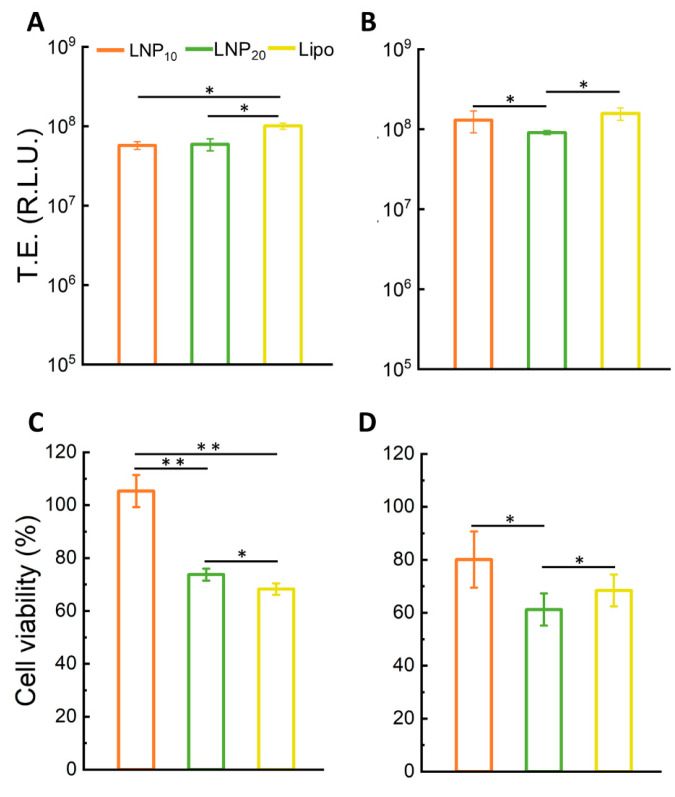
**Transfection efficiency and cell viability of LNPs**. Transfection efficiency (TE) of LNP_10_ and LNP_20_ expressed as relative light units (RLU) for HEK-293 (**A**) and CHO (**B**) cells. Cell viability of HEK-293 cells (**C**) and CHO cells (**D**) after treatment with LNP_10_ and LNP_20_ expressed as a percentage with respect to untreated cells. Statistical significance was evaluated using Student’s *t*-test: * *p* < 0.05; ** *p* < 0.01 (no asterisk means lack of significance). Lipofectamine^TM^ 3000 was used as a control.

The presence of Bragg peaks is due to the lamellar periodicity of the system along the normal direction to the lipid bilayer. The length d of a single repeating unit in the lattice can be calculated as d = 2π/q_001_ = 6.9 nm and corresponds to the sum of the bilayer thickness (d_B_) and the thickness of the water/DNA layer (d_W_). More repeating units constitute a domain. By employing the Debye–Scherrer relation, the average domain size L_m_ was measured, i.e., L_m_ = 2π/Δq_001_, where Δq_001_ is the full width at half maximum of the first-order Bragg peak. We obtained L_m_ = 42.7 nm, which corresponds to multiple short-range domains made of *n* = L_m_/d ≈ 6 repeating units. These results are in agreement with previous findings, according to which the internal structure of the LNPs is due to the arrangement of locally ordered domains along the normal direction to the lipid bilayer but randomly oriented along one particle radius [10] (Figure 3B, inset). This nanoscale arrangement of LNPs is less ordered than that of multilamellar “onion-like” lipoplex [31] complexes made of the same lipid species and also at the same lipid molar ratio and lipid/DNA ratio. Another distinctive feature of the SAXS pattern of the LNPs is the absence of the broad diffraction peak that is typically observed in the SAXS pattern of lipoplexes. That peak, referred to as the “DNA peak” [32], is usually located between the (*001*) and the (*002*) Bragg peaks and arises from a one-dimensional in-plane DNA–DNA lattice. Its absence in the SAXS trace of Figure 3B suggests that DNA is less densely packed in LNPs than in lipoplexes. This less ordered nanoscale organization may be more easily disintegrated upon interaction with cellular membranes and could contribute to explaining the disassembly ability of the LNPs [33]. The next step was the in vitro validation of LNP_10_ and LNP_20_. To this end, we used human embryonic kidney 293 (HEK-293) and Chinese hamster ovary (CHO) cell lines that are frequently used in biological research. As the reproducibility of experimental data is a critical issue affecting the inconsistency of TE reports, user variability was assessed first. Results reported in Figure S2 in the Supplementary Materials demonstrated the high reproducibility of TE data. Figure 4 displays TE and cell viability of LNP_10_ and LNP_20_ in HEK-293 and CHO cells. The LNP formulations were as efficient as Lipofectamine^TM^ 3000 in transfecting both cell lines. These findings are remarkable since Lipofectamine is the gold standard of transfection reagents with exceptional TE due to its virus-like intracellular trafficking behavior [25]. As the next step, LNPs were administered to HEK-293 and CHO cells and the cell viability was measured after 48 h. LNP_10_ was found to have a minor effect on cell viability, while LNP_20_ produced significant cytotoxicity (Figure 4C). The higher cytotoxicity of LNP_20_ than LNP_10_ is likely related to its higher dose of cationic lipids that can cause several changes to cells, such as cell shrinking, reduced number of mitoses, and vacuolization of the cytoplasm [34].

**Figure 5:**
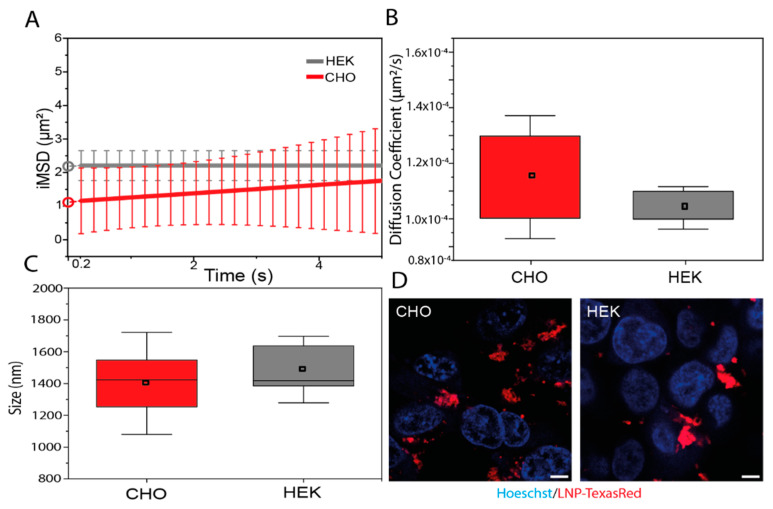
**Intracellular behavior of LNPs**. (**A**) Average iMSD curves of LNP–Texas Red in CHO (red, *n* = 6) and HEK cells (grey, *n* = 6). *y*-Axis intercept derived by fitting (circles) represents the average dimension of LNP–Texas Red clusters adhering to the plasma membrane. (**B**) iMSD-derived diffusion coefficients of LNP–Texas Red in CHO (red) and HEK-293 (grey). Boxes represent 25th–75th percentiles; whiskers represent standard deviation. (**C**) iMSD-derived size of LNP–Texas Red clusters in CHO (red) and HEK-293 (grey). Boxes represent 25th–75th percentiles; whiskers represent maximum–minimum ranges; lines represent median values. (**D**) Exemplary images of CHO and HEK-293 cells labeled with Hoechst (blue, for nuclei) and incubated with LNP–Texas Red (red). Scale bar = 5 μm.

According to TE and cell viability results, LNP_10_ was selected as the best candidate for the delivery of a DNA vaccine. The chemical–physical stability of LNP_10_ was assessed for up to 15 days through DLS measurements. In this timescale, the Z-potential of LNP_10_ fluctuated between 35 and 45 mV, whereas its size increased from 160 to 240 nm (Figure S3A). This resulted in a slight decrease in the transfection efficiency (Figure S3B). To better elucidate the transfection behavior of LNP_10_, we performed fluorescence confocal microscopy analysis of its uptake in cells (Figure 5). Texas Red–labeled LNP_10_ were administered to HEK-293 and CHO cells to study their structural and dynamics properties using spatiotemporal correlation spectroscopy in the form of imaging-derived mean square displacement (iMSD) analysis, a technique capable of providing the average diffusion coefficient and dimension of fluorescent LNPs [17,18]. From fitting the iMSD curves (Figure 5A), the retrieved diffusion coefficient was found to be similar in CHO and HEK-293 cells and with an absolute value (i.e., ~10^−4^ μm^2^/s) that describes quite immobile structures with respect to other cytoplasmic organelles (Figure 5B). Similarly, the average size of the Texas Red–LNP_10_, calculated by the *y*-axis intercept of iMSD curves, is comparable between the two cell lines (Figure 5C) and corresponds to micrometric aggregates distributed near the cell membranes in both cases (Figure 5D). This evidence, in addition to being in accordance with the low diffusivity described above, is in keeping with data collected on other lipid-based systems with high fusogenic properties [35].

To assess the ability of LNP_10_ to deliver cancer vaccines, we prepared a variant of LNP_10_ encapsulating pVAX-hECTM, a DNA vaccine conceived against the oncogene HER2 and known to be able to elicit a protective immune response against HER2-positive breast cancer in preclinical models [15]. HER2 is a tyrosine kinase receptor overexpressed in roughly 20% of breast cancer patients and correlates with poor prognosis [36]. HER2 is considered an optimal target for cancer immunotherapies since it is expressed on the cell membrane, and thus it can be targeted by antibodies and cell-mediated immunity. Antibodies can directly inhibit the signaling pathways downstream HER2 or mediate indirect reactions, such as antibody-dependent cell and complement-mediated cytotoxicity. The advent of the HER2 monoclonal antibody trastuzumab improved the overall survival and time-to-disease progression of patients with HER2-positive breast cancer [37]. However, many patients do not benefit from treatment because of therapy resistance. In this scenario, anti-HER2 DNA vaccination represents a promising alternative strategy, but its clinical efficacy needs to be improved. The optimization of DNA vaccine delivery systems represents a key point to address. LNPs can improve the immunogenicity of the carried DNA vaccines and facilitate their administration. HER2 comprises an extracellular (EC) domain of 654 amino acids that contains four subdomains (I/L1, II/CR1, III/L2, and IV/CR2), a single transmembrane-spanning domain (TM), and a long cytoplasmic tyrosine kinase domain (IC). pVAX-hECTM encodes a truncated version of the human HER2 protein, encoding the EC and TM domains but lacking the IC domain [15].

**Figure 6:**
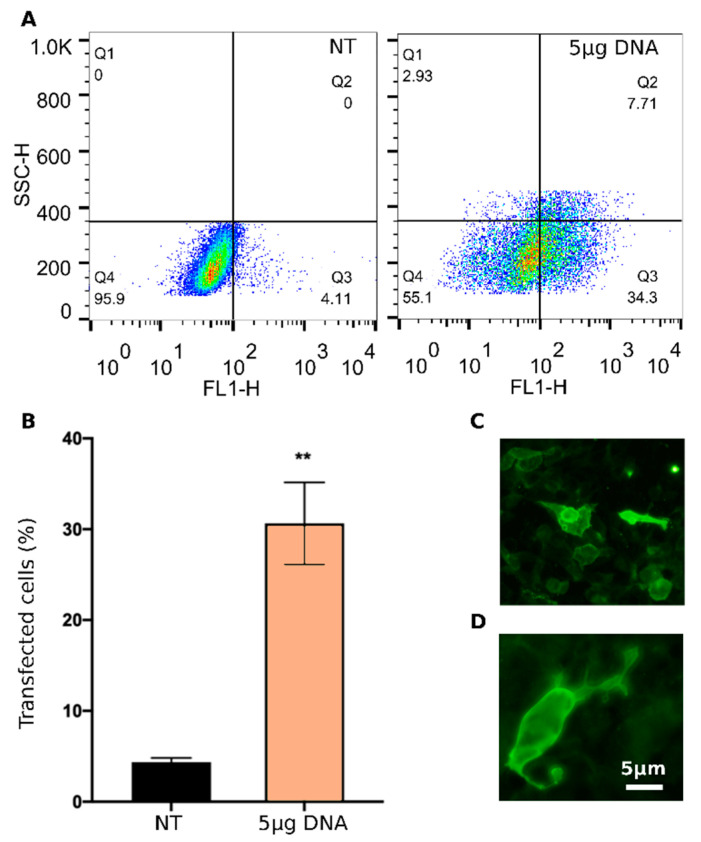
**Transfection efficiency of LNP_10_ carrying the anti-HER2 DNA vaccine pVAX-hECTM**. (**A**) Representative dot plots (increasing dot density from blue to red) and (**B**) bar graphs showing the percentage of HER2-positive HEK-293 cells at 48 h post-transfection with pVAX-hECTM encapsulated into LNP_10_ (5 μg DNA/well) in comparison with non-treated (NT) cells and analyzed by flow cytometry. Error bars represent average ± S.D. (*n* = 6) ** *p* < 0.001 unpaired *t*-test. (**C**,**D**) Fluorescence microscope photographs of HEK-293 cells at 48 h post-transfection with pVAX-hECTM delivered by LNP_10_ (10× and 40× magnifications in C and D, respectively). (NT: non-treated cells).

To assess the ability of LNP_10_ to deliver DNA vaccines, HEK-293 cells were transiently transfected with pVAX-hECTM encapsulated into LNP_10_, and the expression of the encoded HER2 antigen was verified first by flow cytometry (Figure 6A,B) and then by immunofluorescence assay (Figure 6C,D) using the anti-human HER2 monoclonal antibody trastuzumab.

FACS analysis demonstrates that LNP_10_ efficiently delivers pVAX-hECTM into HEK-293 cells, leading to 30% transfected cells. The immunofluorescence assay confirms that HEK-293 cells were able to ectopically express the target antigen after transfection by LNP_10_. In particular, the strong membrane fluorescence signal observed in the transfected HEK-293 cells indicates that the HER2 antigen is exposed at the cell membrane as required for the induction of a protective antibody response [38].

## 4. Conclusions

Technological issues have limited the development of DNA vaccines for cancer so far. LNPs may help to address these issues and are now considered one of the most advanced delivery technologies. Using a screening strategy based on the physical–chemical characterization, and in vitro validation, we developed an LNP formulation with a distinct ability to transfect cell model lines as efficiently as Lipofectamine^TM^ 3000. A variant of this formulation loaded with a DNA vaccine conceived against the oncogene HER2 produced a massive expression of the HER2 antigen on the cell membrane of the HEK-293 cells. Our results provide new insights into the structure–activity relationship of DNA-loaded LNPs and may contribute to lifting this gene delivery technology from basic knowledge to preclinical studies.

## Figures and Tables

**Table 1:** **Lipid composition of LNPs**. Quantity of lipids expressed as a molar percentage for each LNP formulation and respective total lipid/pDNA weight ratios (Rw).

	DOTAP (%mol)	Dc-Chol (%mol)	DOPE (%mol)	DOPC (%mol)	Rw
LNP_5_	25	25	25	25	5
LNP_10_	25	25	25	25	10
LNP_20_	25	25	25	25	20

## Data Availability

Data reported in this study are available on request to the corresponding authors.

## Supplementary Materials

The following supporting information can be downloaded at: https://www.mdpi.com/article/10.3390/pharmaceutics14081698/s1. Table S1: Size, zeta-potential, and polydispersity index (PdI) of LNP_5_, LNP_10_, and LNP_20_. Figure S1: (A) Calibration curve (black points) obtained through fluorescence signal measurement of pDNA and the related fluorescence signals obtained from LNP_10_ and LNP_20_ before and after treatment with Triton X-100 for measuring non-encapsulated pDNA and total pDNA amounts. (B) Encapsulation efficiency of pDNA within LNP_10_ and LNP_20_ calculated as reported in the Materials and Methods section. Figure S2: (A) Transfection efficiency (TE) of LNP_10_, LNP_20,_ and Lipofectamine^TM^ 3000 was measured by two users (indicated as “User 1” and “User 2”) in independent experiments. (B) High reproducibility of transfection data was demonstrated by linear regression of measured TE values. Figure S3: (A) Size (black) and Z-potential (grey) of LNP_10_ measured at different time points (0, 5, 10, and 15 days). (B) Transfection efficiency (TE) of LNP_10_ on HEK-293 cells measured at 0 and 15 days, expressed as relative light units (RLU) per mg of proteins.
