# Supplementary material for: Efficient Delivery of DNA Using Lipid Nanoparticles
Source: Pharmaceutics. 2022 Aug 15;14(8):1698. doi: 10.3390/pharmaceutics14081698 (PMC9416266; doi:10.3390/pharmaceutics14081698)
Supplement: Supplementary file 1 [file pharmaceutics-14-01698-s001.zip › pharmaceutics-1830107-supplementary.pdf]

pharmaceutics

## Supplementary Materials

**Table S1.** Size, zeta-potential, polydispersity index (PdI), and encapsulation efficiency of $LNP_5$, $LNP_{10}$ and $LNP_{20}$.

| | $LNP_5$ | $LNP_{10}$ | $LNP_{20}$ |
|---|---|---|---|
| **Size (d.nm)** | 451.9 ± 364.1 | 122.6 ± 4.095 | 129.2 ± 3.39 |
| **PdI** | 0.994 ± 0.011 | 0.124 ± 0.002 | 0.268 ± 0.007 |
| **Zeta potential (mV)** | 11.11 ± 8.8 | 47.4 ± 7.55 | 49.4 ± 4.7 |
| **Encapsulation Efficiency (%)** | n.d. | 80.3 | 67.4 |

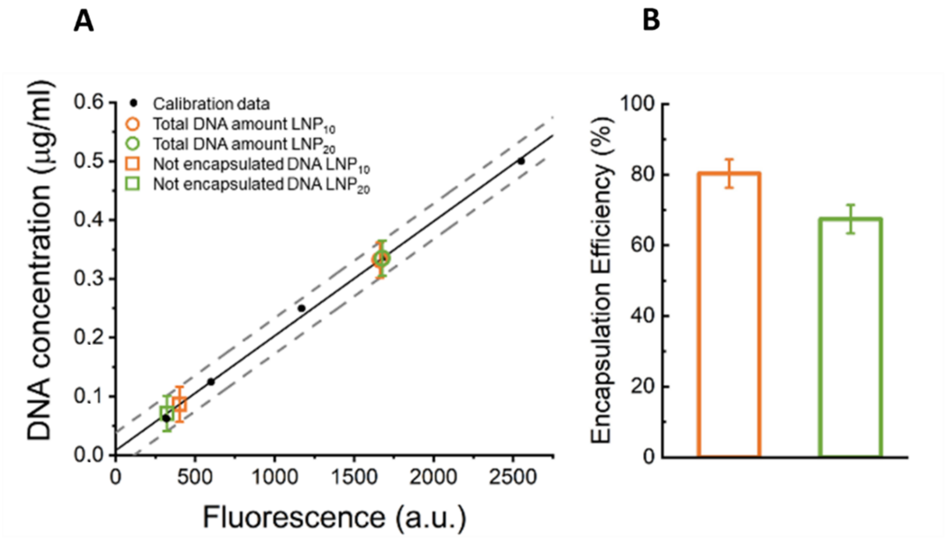


**Figure S1.** (**A**) Calibration curve (black points) obtained through fluorescence signal measurement of pDNA and the related fluorescence signals obtained from $LNP_{10}$ and $LNP_{20}$ before and after treatment with Triton X-100 for measuring non-encapsulated pDNA and total pDNA amounts. (**B**) Encapsulation efficiency of pDNA within $LNP_{10}$ and $LNP_{20}$ calculated as reported in the Materials and Methods section.

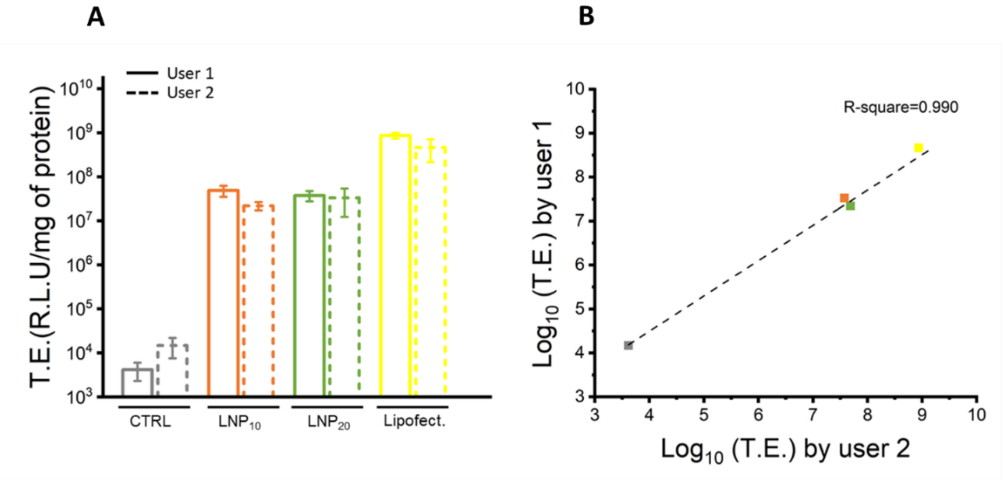


**Figure S2.** (**A**) Transfection efficiency (TE) of $LNP_{10}$, $LNP_{20,}$ and Lipofectamine™3000 was measured by two users (indicated as "User 1" and "User 2") in independent experiments. (**B**) High reproducibility of transfection data was demonstrated by linear regression of measured TE values.

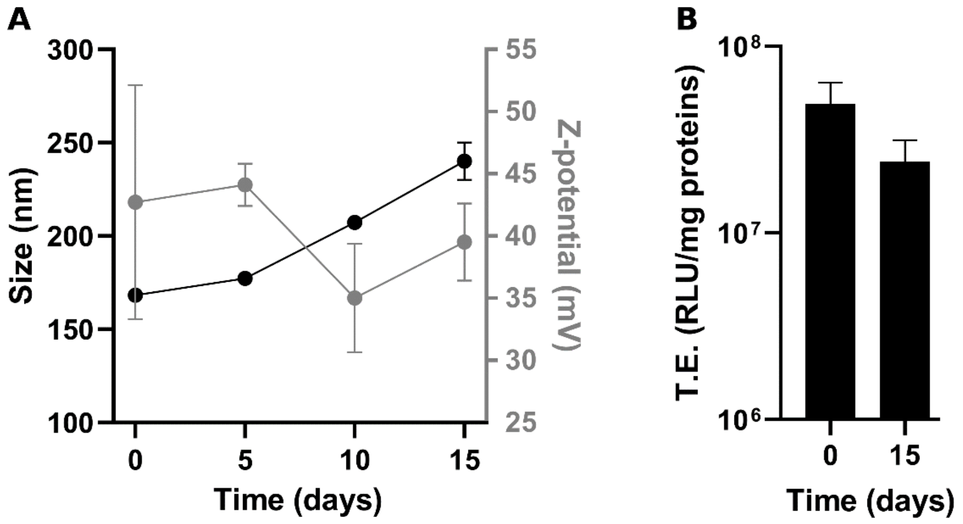


**Figure S3.** (**A**) Size (black) and Z-potential (grey) of $LNP_{10}$ measured at different time points (0, 5, 10, and 15 days). (**B**) Transfection efficiency (TE) of $LNP_{10}$ on HEK-293 cells measured at 0 and 15 days expressed as relative light units (RLU) per mg of proteins. .
